# FOCAL: an experimental design tool for systematizing metabolic discoveries and model development

**DOI:** 10.1186/gb-2012-13-12-r116

**Published:** 2012-12-13

**Authors:** Christopher J Tervo, Jennifer L Reed

**Affiliations:** 1 Department of Chemical and Biological Engineering, University of Wisconsin - Madison, WI 53706, USA

## Abstract

Current computational tools can generate and improve genome-scale models based on existing data; however, for many organisms, the data needed to test and refine such models are not available. To facilitate model development, we created the forced coupling algorithm, FOCAL, to identify genetic and environmental conditions such that a reaction becomes essential for an experimentally measurable phenotype. This reaction's conditional essentiality can then be tested experimentally to evaluate whether network connections occur or to create strains with desirable phenotypes. FOCAL allows network connections to be queried, which improves our understanding of metabolism and accuracy of developed models.

## Background

There are currently over 3,000 completely sequenced bacterial genomes [1]. For many of these sequenced organisms we know relatively little about them compared to well-studied organisms [2], even though they are important for biomedical, environmental, and biotechnological applications. However, their sequenced genomes provide a wealth of data that can be mined to discover their metabolic capabilities and transcriptional regulatory control mechanisms. Knowing how an organism metabolizes compounds, generates energy, produces cellular components, and synthesizes useful products is critical for enhancing chemical production, identifying new drug targets, or improving bioremediation. If little is known about an organism's metabolism and regulation a logical question is where to begin? Moreover, what sets of experiments should one perform to effectively determine how cells utilize and control metabolism?

Mathematical representations of genome-scale networks - known as genome-scale models (GEMs) - enable a quantitative and systematic approach to address this issue. By developing GEMs, the microbial reaction networks can be interrogated to predict growth phenotypes, guide metabolic engineering strategies, elucidate network components and interactions, and facilitate hypothesis-driven discovery [3-6]. However, the successful application of *in silico *metabolic and regulatory models depends on their ability to capture the underlying characteristics of the biochemical networks in the microbe of interest. With increasing improvements in genome sequencing technologies and annotation, and in metabolic network reconstruction [7], the ability to construct GEMs has become more high-throughput. Many of these annotation-derived GEMs possess reactions whose inclusion is based solely on homology or on reproducing growth phenotypes (that is, enabling biomass production); consequently, verifying the metabolic networks derived from genomic data is becoming increasingly important. Without an accurate representation of the microbial network, model driven design of therapeutics and metabolic engineering strategies will be potentially flawed and substantial time and resources may be wasted. Unfortunately, reactions and gene-protein-reaction (GPR) associations can be incorrectly included or omitted during model development due to database, sequencing, and annotation errors, as well as unknown enzyme functionality [4]. Existing models for *Escherichia coli *have been painstakingly developed and refined over the past 20 years, using analysis of experimental data acquired over the past 50 years from hundreds of laboratories. Spending this level of time, effort, and resources to obtain a good understanding of metabolism for every microbial organism of interest is simply intractable. Thus, to streamline the process of model curation, future experiments should be designed to reduce experimental efforts while still effectively probing the biological system of interest. Having the ability to quickly design experiments to test reactions is critical for improving the accuracy and utility of genome-scale models, particularly for less-characterized microorganisms where existing experimental data are limiting.

A GEM can be refined when discrepancies are found between model predictions and experimental observations. Several automated computational approaches have been developed to suggest model improvements based on such discrepancies between model predictions and existing experimental data. Constraint-based model refinement algorithms, such as OMNI, SMILEY, GrowMatch, and GeneForce [8-11], work to improve a model's ability to reflect known experimental results. Depending on the algorithm, this may be accomplished by adding or removing network reactions, modifying GPR associations, modifying biomass compositions or relaxing regulatory rules. These methods successfully improve model accuracy; however, they all rely on available experimental data to first identify model inaccuracies.

Currently, there are no constraint-based methods to efficiently design new experiments to test the accuracy of a given genome-scale metabolic model, and its associated metabolic network reconstruction. To address this limitation, we sought to develop an approach that would identify media and gene knockout conditions under which a chosen reaction is essential for some measurable phenotype (for example, growth). A prior study has used minimal cut sets (MCSs) to identify minimal sets of reactions that if deleted will disable growth [12], and once enumerated MCSs could be evaluated to find a MCS involving the chosen reaction. However, identifying these sets requires computation of elementary modes, and so it can not be applied to genome-scale networks, which often contain approximately 500 to 2,000 reactions [13]. Flux balance analysis (FBA) [6] can be used to predict if a reaction is essential for growth in genome-scale networks; however, finding conditions under which a chosen reaction is essential may require an exhaustive search of multiple gene knockout combinations. Additionally, since FBA predictions and MCSs are condition-specific, these methods would need to be evaluated in all possible media combinations, making the task even more computationally challenging.

To address this experimental design challenge, we used concepts from flux coupling analysis to efficiently identify media and knockout conditions under which a chosen reaction is required to enable flux through another experimentally measurable reaction (for example, growth). Flux coupling analysis characterizes the relationships between reactions in a fixed network [14], and has been used to investigate gene regulation and gene essentiality [15,16], and for metabolic flux analysis [17,18]. In flux coupling analysis, all reversible reactions are first decoupled into a forward and reverse reaction. Then, the maximum and minimum flux ratio between two reactions is calculated and used to characterize the relationships between fluxes (v) through these two reactions. For example, if the minimum flux ratio (v_chosen_/v_measured_) is positive, then it implies that a chosen flux, v_chosen_, must be non-zero if another experimentally measurable flux, v_measured_, is non-zero (v_measured _→ v_chosen_). For our purposes, reactions are considered coupled if the minimum flux ratio is positive or the maximum flux ratio is a finite number; otherwise, they are uncoupled. These reaction couplings are highly dependent on the network and the environmental conditions used [14] and so flux coupling analysis has to be reapplied if the network changes (for example, a gene or reaction is deleted or added), or a different experimental condition is used (for example, glucose versus xylose media). As such, flux coupling analysis cannot identify network or environmental changes that lead to coupling between a chosen flux and an experimentally measurable flux. Thus, we developed the forced coupling algorithm (FOCAL) that will identify media conditions and gene deletions (which together form the coupling conditions) such that chosen fluxes are coupled with some measurable flux (that is, a flux that can be measured directly in experiments). Under these conditions, flux through a measurable reaction (for example, biomass production or by-product secretion) requires flux through one or multiple chosen reaction(s), and we refer to these conditions identified by FOCAL as coupling conditions.

By finding coupling conditions in which biomass production depends on flux through a chosen reaction(s), we can design new growth phenotyping experiments to detect whether a chosen reaction occurs by simply monitoring cellular growth. Experimentally testing these coupling conditions allows for a variety of interesting conclusions to be made about the metabolic network. First, if no growth under the proposed coupling conditions occurs, then there is a problem with the model. In this case it is possible that the chosen reaction does not occur because the associated enzyme is not expressed under this condition (due to regulation) or that the enzyme does not catalyze the reaction of interest (incorrect annotation). This means that regulatory, reaction and/or GPR changes are needed to correct the model. Second, if the chosen reaction is found to be conditionally essential under the coupling condition (meaning growth occurs under the coupling condition but when the chosen reaction is additionally eliminated no growth occurs), then the chosen reaction and its associated GPR relationships appear to be correct within the model. Third, if the chosen reaction is not conditionally essential under the coupling condition, then components (for example, reactions or isozymes) are missing from the network and can be suggested using computational approaches [8,9].

**Figure 1:**
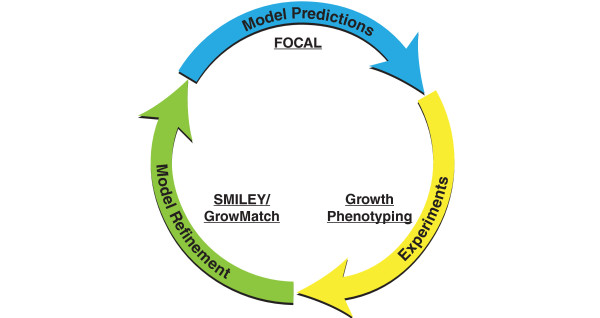
**FOCAL refinement cycle**. The model testing and refinement cycle is a three part process. First, FOCAL is used to design experiments where a particular reaction should be essential. The necessary mutants and media are prepared and growth phenotype experiments are performed. If any discrepancies are observed, the errors are corrected using various methods to suggest model improvements. These modifications can subsequently be tested further by designing new FOCAL designed experiments based on the refined model.

A cycle of model testing and improvement can be established by iteratively using FOCAL to design experiments, conducting the FOCAL designed experiments, and adjusting the model when discrepancies between model predictions and experimental results are found (Figure 1).

By enumerating and testing such coupling conditions, it is possible to not only confirm the presence of existing network components and interactions, but also to discover new interactions within the cellular network when the experimental results do not agree with model predictions. Additionally, since GEMS are powerful tools for enhancing biochemical production [19], we have also used FOCAL to design strains with complex and atypical phenotypes, such as the concurrent utilization of multiple substrates by a single strain. By combining our novel experimental design algorithm with existing approaches for refining models [8-10], we envision an integrated computational and experimental platform (Figure 1) will be established that enables rapid development of highly accurate models and improved understanding of microbial metabolism across a wide variety of organisms, including those that are not well characterized experimentally.

## Results and discussion

FOCAL builds on concepts from the flux coupling framework [14], where the latter is capable of determining the relationships between two reaction fluxes given a fixed network and environment. Unlike the flux coupling framework, FOCAL actively works to create coupling within a network by selecting genetic and environmental conditions such that flux through a particular reaction (v_chosen_) becomes essential for another measurable flux (v_measured_). While a variety of different types of flux coupling relationships exist [14], FOCAL looks specifically for circumstances under which the existence of a particular measurable flux, v_measured_, implies the existence of flux through another reaction, v_chosen _(and, from contraposition, no flux through v_chosen _implies no flux through v_measured_). Here, we discuss FOCAL's proposed solutions for coupling reactions to biomass in four genome-scale metabolic models. Using these results, we illustrate FOCAL's utility for systematically evaluating and refining metabolic models by comparing FOCAL predictions to new and existing experimental results. We further show how FOCAL led to the discovery of a new isozyme (YeiQ) for two reactions in glucuronate and galacturonate catabolism. Finally, we demonstrate the use of FOCAL to design more complex phenotypes, such as mutants that must concurrently utilize glucose and xylose in order to grow.

### Forced coupling algorithm: an illustrative example

**Figure 2:**
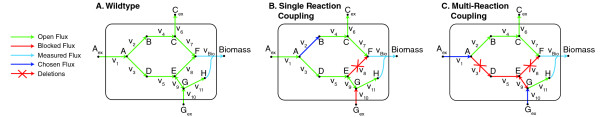
**An illustrative example of FOCAL**. FOCAL is first used to couple cellular growth (v_bio_) with a chosen reaction flux (v_2_). **(a) **In the uncoupled system, v_4 _is coupled with v_2 _(that is, v_4 _≥ 0 implies v_2 _≥ 0) but v_bio _is not coupled with v_2_. **(b) **In the coupled case, v_bio _is coupled with v_2 _(v_bio _**→ **v_2_). Here, metabolite A_ex _is the only nutrient (no G_ex_), and a gene associated with v_8 _is deleted such that the upper pathway is required to synthesize metabolite F. Under these circumstances, flux through v_bio _requires flux through v_2_. Moreover, removal of v_2 _(along with v_8_) will result in a non-viable cellular mutant. **(c) **FOCAL can also be used to create substrate co-utilizing mutants where deletion of v_3 _and v_8 _requires the co-utilization of metabolites A and G in order to produce both biomass components, F and H.

Using a small reaction network, we will first demonstrate how FOCAL works and how to interpret its results (Figure 2). FOCAL proposes minimal media components and knockout mutations (if needed) such that flux through the chosen reaction is required for biomass production. In the first example, FOCAL's objective is to design an experiment to test if the v_2 _flux occurs. In the wild-type network (Figure 2a), biomass production (v_bio_) and v_2 _are uncoupled due to alternative ways of making the two biomass components, F and H (for example, using v_3 _or, if metabolite G_ex _is in media, v_10_). FOCAL indicates that coupling between v_2 _and v_bio _(v_bio _→ v_2_) can be obtained by using metabolite A as the sole minimal media component and deleting genes associated with v_8 _(Figure 2b). FOCAL can also be extended to design substrate co-utilizing mutant strains as shown in Figure 2c. To accomplish this, FOCAL looks for coupling conditions composed of minimal media specifications and gene deletions so that multiple reactions, in this case substrate transporters (v_1 _and v_10_), are required in order for the cell to grow (v_bio _→ v_1 _and v_10_). To achieve this, FOCAL recommends deleting genes associated with v_3 _and v_8 _and using both metabolites A and G in the minimal media. The resulting mutant requires both metabolites A and G to produce biomass components F and H, respectively. In some instances, alternative FOCAL solutions will exist and these can be found using additional integer cut constraints (see Materials and methods for details).

### Application to genome-scale metabolic networks

**Figure 3:**
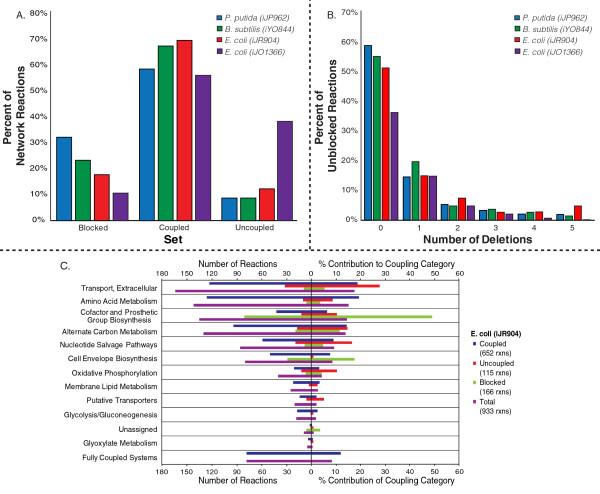
**Various FOCAL statistics for genome-scale models**. **(a) **Percentage of blocked, coupled, and uncoupled network reactions for each model evaluated. **(b) **Percentage of unblocked reactions from each model that require 0 to 5 deletions to become coupled with biomass. Reactions with zero gene deletions can be coupled solely by modifying the media composition. For all models, except iJO1366, the number of deletions is the number of necessary gene deletions. For iJO1366, additional isozyme deletions may be necessary (the total number of gene deletions needed for iJO1366 can be found in Figure S3 in Additional file 2). **(c) **Distribution of iJR904 reactions belonging to a given coupling category (coupled, uncoupled or blocked) across metabolite subsystems. The percentage (left) or number (right) of reactions within a given coupling category that belong to a particular subsystem is shown. The fully coupled metabolic subsystem in (c) is composed of metabolic subsystems in which all reactions could be coupled to biomass, and contains the citric acid cycle, pentose phosphate cycle, nitrogen, pyruvate and methylglyoxal metabolism, purine and pyrimidine biosynthesis, anaplerotic, and putative reaction pathways.

To determine sets of experiments to test for all metabolic reactions in a network, FOCAL was applied to every reaction present in genome-scale metabolic networks for *Escherichia coli *[20,21], *Bacillus subtilis *[22] and *Pseudomonas putida *[23] using biomass (that is, growth) as v_measured_. For each model, we specified sets of selectable carbon sources, nitrogen sources, electron acceptors, and additional nutrients that can be used to compose the minimal media (Additional file 1) and additional algorithm parameters (for example, maximum number of deletions; see Materials and methods for details). Based on FOCAL results, the reactions in these networks were categorized as coupled (a coupling condition could be found by FOCAL), uncoupled (no coupling condition could be found) or blocked (a reaction is incapable of carrying flux when all possible nutrients are provided) (Figure 3a). Each FOCAL proposed strategy was further evaluated based on the number of gene deletions required to achieve the desired reaction coupling between a metabolic reaction and biomass production (Figure 3b). Across the four models, a coupling condition was found for approximately 60 to approximately 85% of the unblocked reactions, and approximately 35 to 60% of these cases did not require any gene deletions, indicating that the media conditions alone were enough to couple the reaction to biomass (common deletions for each model can be found in Table S1 in Additional file 2). For the iJR904 *E. coli *network, we also assessed how these reaction categorizations (that is, coupled, uncoupled, and blocked) were distributed across different metabolic subsystems (Figure 3c) and how media components were used (Figure S1 in Additional file 2). In *E. coli*, the cell envelope biosynthesis and the cofactor and prosthetic group biosynthesis subsystems contain a disproportionate number of blocked reactions. This is mainly due to the absence of many cofactors and prosthetic groups in the biomass reaction. Transporter, nucleotide salvage and oxidative phosphorylation reactions were the most difficult to find coupling conditions for, which may be attributable to redundant pathways, multi-functional enzymes, multiple isozymes or FOCAL simulation parameters. For *E. coli*, glucose, ammonia, and oxygen were the most frequently used carbon, nitrogen and electron acceptors utilized. Interestingly, the additional nutrients used in FOCAL designed experiments for *E. coli *and *B. subtilis *were quite different (Figure S2 in Additional file 2), likely due to differences in transporters between the two models.

We also investigated if the gene deletions selected by FOCAL for the iJR904 *E. coli *network were close to the chosen reaction that becomes coupled with biomass. The shortest path distance between deleted reactions found by FOCAL and the chosen reaction was calculated for all proposed gene deletions associated with a single reaction (see Additional file 2 for details). For both a directed and undirected version of the metabolic network, the reactions that FOCAL deletes to achieve coupling tend to be closer on average (2.80 for the directed and 2.42 for the undirected network) than would be expected if deletions were selected randomly (4.99 and 3.90 for the directed and undirected network, respectively; in both cases *P*-value <1e-10 using one-tailed *t*-test).

Further analysis was done to investigate why FOCAL could not find coupling conditions for the 115 reactions in the iJR904 *E. coli *network that could not be coupled to biomass. These 115 reactions were subsequently re-evaluated with FOCAL using a higher gene deletion limit (up to 10 gene deletions), adding more measurable reactions that FOCAL could use as v_measured _besides biomass production (by expanding the *Coupling *set, described in Materials and methods), and expanding the list of additional nutrients. With these three changes, approximately a third of the previously uncoupled reactions were coupled by FOCAL to a measurable flux (Table 1). The remaining reactions could not be coupled for a variety of reasons. Around 40% of uncoupled reactions were involved in highly robust and interconnected pathways where reactions are catalyzed by the same multifunctional enzyme. For example, six reactions in the nucleoside salvage pathway (NTPP1-3 and NTPP5-7) dephosphorylate nucleosides and are all catalyzed by MazG, making it difficult to find coupling conditions that force one reaction to be essential while producing a viable mutant. Additionally, some reactions (approximately 10% of uncoupled reactions), based on a directed shortest path analysis, were not connected to biomass. For the remaining reactions, coupling conditions do not exist because they are involved in recycling metabolites, only participate in futile cycles, or have alternative reactions that cannot be eliminated due to their GPR relationships (see Figure S4 in Additional file 2 for examples).

**Table 1:** Comparison of FOCAL results for iJR904 and mutant aerobic growth phenotypes

Category	Frequency	Percentage of uncoupled
**Can be coupled using:**		
More deletions	8	7.0
More measurable fluxes	8	7.0
More deletions/ measurable fluxes	8	7.0
Expanded additional nutrient set	14	12.2
		
**Still cannot be coupled because:**		
Highly robust/connected	48	41.7
No connection to biomass	11	9.6
Other reasons	18	15.7
		
**Total**	115	100

Compared to the smaller *E. coli *model (iJR904), coupling conditions were found for a lower percentage (approximately 60%) of the unblocked reactions in the most recent *E. coli *model (iJO1366) [21]. We further investigated why coupling conditions could not be found for a larger fraction of these iJO1366 reactions, many of which involved transporters and membrane lipid metabolism (40% of all uncoupled reactions; Figure S3 in Additional file 2). In many cases, no coupling conditions exist due to the presence of alternative reactions that are not associated with genes (for example, transporters like XANt2pp and XANtpp) or are associated with the same genes or essential genes. Each of the 24 EAR reactions, for example, has an alternative reaction that uses a different cofactor (NADPH versus NADH) and is associated with the same protein (FabI). As a result, the alternative reactions cannot be eliminated without also eliminating the chosen reaction. Other reactions that recycle metabolites back to their precursors were also in the uncoupled category since the recycling is never essential. The 98 phospholipase and lysophospholipase reactions that degrade phospholipids are examples of these. Another related problem involves the irreversible export of compounds from the cytosol, which prevents their incorporation into biomass (for example, ZN2t3pp and ZN2abcpp), while other reactions cannot be coupled to biomass without adding compounds to the biomass equation. For example, the 14 PSD and PSSA reactions produce phospholipids that are not part of biomass.

Thus, an increased number of alternative reactions, recycling reactions and multifunctional enzymes in iJO1366 reduces the number of reactions that can be coupled to biomass. As such, the increase in uncoupled reactions is not a failing of FOCAL, but rather a feature of the more comprehensive network. Future research could look to overcome this by instead generating coupling conditions for genes rather than reactions; in this way conditionally essential genes could be identified that would indicate that some of these uncoupled reactions take place. Additionally, while manual efforts were used to identify why particular reactions cannot be coupled to biomass, this process could be semi-automated, by identifying clusters of reactions that share common genes and by determining cycles in metabolism (see Additional file 2 for details).

### Comparison of FOCAL predictions to experimental results

FOCAL coupling conditions for *E. coli *iJR904 reactions associated with a single gene and involving only media specifications (that is, without requiring any gene deletions) were compared to previous studies where *E. coli *single knockout strains were tested for aerobic growth in glucose [24] and glycerol [25] minimal medium (Table 2). These experimental results were used to verify the conditional essentiality of the 232 single-gene reactions FOCAL coupled to biomass under these same media conditions. For the glucose experiment, a mutant was considered not to grow if the optical density (OD) at 24 and 48 hours was less than 0.10. For the glycerol aerobic experiment, we used the same growth classification as reported previously [25]. Of the 232 single-gene reactions that are coupled with biomass under these two conditions, experimental data were only available for 178 of the related mutants, and of these, 152 (approximately 85%) were conditionally essential, meaning that mutants missing these chosen reactions were unable to grow specifically under the proposed FOCAL media condition (Table 1). Of the 26 model-data discrepancies, 2 mutants (Δ*aroD *and Δ*nadC*) were shown to be unable to grow on glucose in other experiments [26] and another 2 mutants (Δ*folB *and Δ*folP*) were shown to have gene duplications [27], indicating these 4 cases are likely not discrepancies. The remaining 22 genes that were not conditionally essential indicate that changes to the model are needed. Model changes based on these datasets have been suggested previously [25] and involve: (1) eliminating components from the biomass equation; (2) accounting for additional transporters; and (3) adding isozymes or alternative reactions. This analysis illustrates how FOCAL results can provide confidence in model content and can lead to suggestions for improving the models when FOCAL predictions do not match experimental results.

**Table 2:** Categorization of initially uncoupled reactions in iJR904

	Glucose	Glycerol
Coupled reactions using only media	193	98
Reactions associated with single genes	134	98
Confirmed conditionally essential genes	108	44
No experimental data	0	54
Percentage agreement	81%	100%

**Figure 4:**
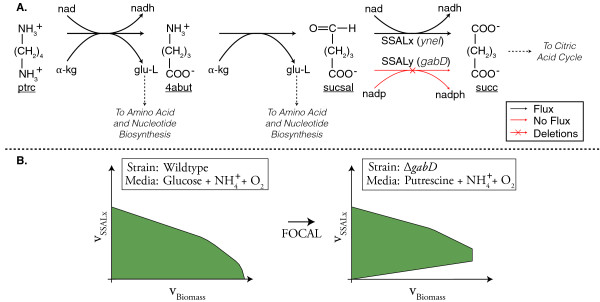
**Application of FOCAL to couple SSALx with biomass production**. **(a) **The putrescine degradation pathway. FOCAL predicts that the NAD-dependent succinic semialdehyde dehydrogenase (SSALx) reaction is coupled with biomass by removing *gabD *(whose gene product catalyzes SSALy) and growing the mutant in minimal media with putrescine and NH_4_^+^. **(b) **The feasible region for the Δ*gabD *mutant under this condition excludes points that lie on the x-axis with the exception of the origin (right), while the wild-type can grow on glucose without flux through SSALx (left). Metabolite abbreviations not reported in the text: α-kg, α-ketoglutarate; glu-L, L-glutamate.

By determining coupling conditions for reactions with unknown GPRs, it is also possible to use FOCAL results to design high-throughput screens to identify genes associated with these so-called orphan reactions. Of the 39 orphan reactions in iJR904 that are not transporters, coupling conditions were found by FOCAL for 27 reactions (Table S2 in Additional file 2). These coupling conditions can potentially be used to screen knockout mutant libraries to find conditionally essential genes that would be candidate genes responsible for these orphan reactions. The NAD-dependent succinic semialdehyde reaction, SSALx (Figure 4), was one such orphan reaction, whose associated gene (*yneI*) has now been identified [28]. FOCAL predicts that the SSALx reaction is required in a Δ*gabD *mutant for aerobic growth on putrescine (ptrc) as a carbon source or on 4-aminobutanoate (4abut) as either a carbon or a nitrogen source. Both ptrc and 4abut are ultimately broken down into succinic semialdehyde (sucsal), which must be subsequently consumed by one of the two succinic semialdehyde dehydrogenases. The Δ*gabD *mutation prevents the NADP-dependent SSALy reaction from occurring and leaves NAD-dependent SSALx reaction as the sole succinic semialdehyde dehydrogenase. Consequently, the Δ*gabD *mutant in one of these ptrc/4abut media conditions must use SSALx to grow and the desired flux coupling is obtained. Experimentally the Δ*yneI*Δ*gabD *double mutant cannot grow during growth on putrescine as a carbon source [29], indicating that FOCAL designed experiments can potentially be used to find genes for orphan reactions.

**Figure 5:**
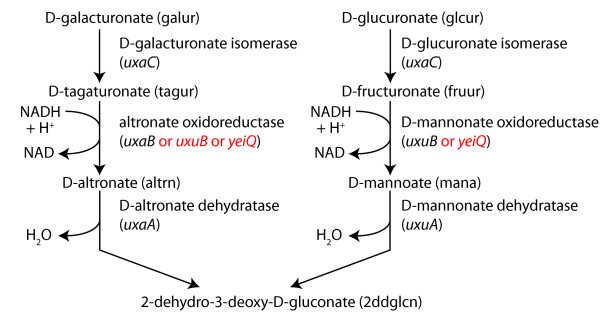
**D-galacturonate and D-glucuronate degradation pathways**. Reactions involved in the degradation of galacturonate and glucuronate. Items in parentheses next to metabolites indicate metabolite abbreviations, and items in parentheses under enzymes indicate the associated genes. Gene names in black are those in the original iJR904 model, while those in red indicate additional functionality discovered by FOCAL designed experiments that are added to the model to recapitulate experimental results.

To further illustrate the use of FOCAL in a model refinement cycle, growth phenotype experiments were performed based on FOCAL results for reactions in alternative carbon metabolism. Reactions involved in galacturonate and glucuronate catabolism (Figure 5) were selected due to the number of experiments proposed by FOCAL using these carbon sources and because reactions in these pathways were coupled to biomass using only media conditions allowing for facile testing (Table 3). All FOCAL predictions were consistent with measured single knockout mutant growth phenotypes (that is, the genes associated with these chosen reactions were conditionally essential as predicted by FOCAL) with the exception of the *ΔuxaB *and *ΔuxuB *mutants, which grew on galacturonate and glucuronate, respectively (Table 4). Since the UxaB and UxuB enzymes carry out similar transformations, we initially hypothesized that the two proteins may be able to catalyze both transformations. However, a double knockout *ΔuxaBΔuxuB *mutant was still able to grow on both carbon sources. A BLASTp search found an oxidoreductase gene with uncharacterized function, *yeiQ*, had significant homology to *uxaB *(E-value = e-21) and *uxuB *(E-value = e-155). Subsequent removal of *yeiQ, uxaB*, and *uxuB *eliminated the ability of strains to grow on glucuronate and galacturonate (Table 4). The results of these additional mutant phenotyping experiments (Table 4; Figure S5 in Additional file 2) suggest that the altronate oxidoreductase reaction could be catalyzed by UxaB, UxuB, or YeiQ and the mannonate oxidoreductase reaction could be catalyzed by UxuB or YeiQ.

**Table 3:** FOCAL designed experiments for reactions in galacturonate and glucuronate catabolism

Chosen reaction^a^	Enzyme	Associated gene	FOCAL selected carbon source
galur tagur	D-Galacturonate isomerase	*uxaC*	D-Galacturonate
h + nadh + tagur altrn + nad	Altronate oxidoreductase	*uxaB*	D-Galacturonate
altrn → 2ddglcn + h_2_o	Altronate hydrolase	*uxaA*	D-Galacturonate
glcur fruur	D-Glucuronate isomerase	*uxaC*	D-Glucuronate
fruur + h + nadh mana + nad	D-Mannonate oxidoreductase	*uxuB*	D-Glucuronate
mana → 2ddglcn + h_2_o	D-Mannonate hydrolyase	*uxuA*	D-Glucuronate

^a^Abbreviations match those shown in Figure 5.

**Table 4:** FOCAL experimental results

	Galacturonate			Glucuronate	
			
Strain	Experimental	Model		Experimental	Model
BW25113	+	+		+	+
*ΔuxaA*	-	-		NA	+
*ΔuxaB*	+^a^	-		NA	+
*ΔuxaC*	-	-		-	-
*ΔuxuA*	NA	+		-	-
*ΔuxuB*	NA	+		+	-
*ΔuxaB ΔuxuB*	+^a^	-		+	-
*ΔyeiQ*	+	+		+	+
*ΔuxaB ΔyeiQ*	+^a^	-		NA	-
*ΔuxuB ΔyeiQ*	NA	+		-	-
*ΔuxaB ΔuxuB ΔyeiQ*	-	-		-	-

^a^Growth was delayed by >48 hours. NA, these experiments were not performed.

### Substrate co-utilization strain designs

**Figure 6:**
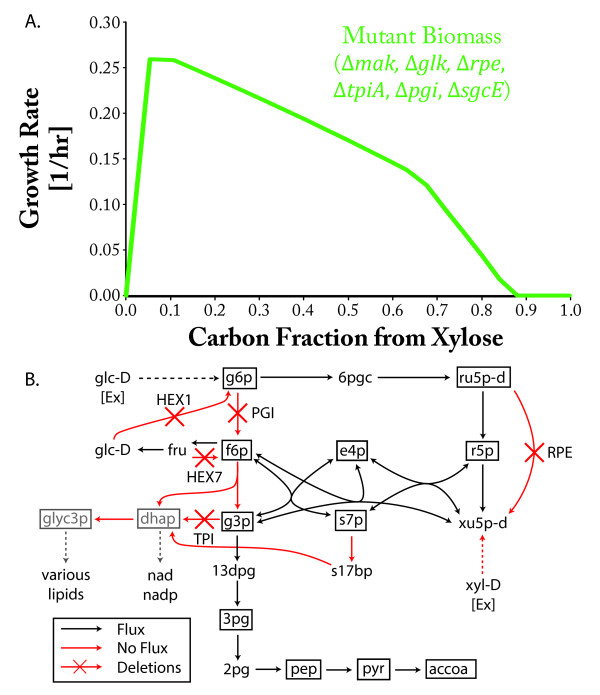
**FOCAL predicted glucose/xylose co-utilization conditions**. **(a) **Maximum FBA predicted anaerobic growth of the FOCAL designed *E. coli *strain as a function of the xylose fraction of the carbon source. The ratio of glucose and xylose within the minimal media was varied while maintaining a constant carbon uptake into the iJO1366 network (110 mmol C·gDW^-1^·h^-1^). Under FOCAL's proposed conditions, the strain is incapable of growth when the media is composed entirely of glucose or xylose due to an inability to produce all biomass components. For comparison, the maximum predicted wild-type rate growth is 0.423 h^-1 ^on pure glucose and 0.362 h^-1 ^for pure xylose (not shown). **(b) **Possible fluxes through central metabolism in the mutant when grown only on glucose. Under these circumstances, the mutant is unable to produce dihydroxyacetone phosphate and glycerol-3-phosphate, which are critical for synthesizing NAD(P) and phospholipids. On xylose only (not shown), the mutant is incapable of sustaining flux beyond the pentose phosphate pathway. Boxed metabolites indicate biomass precursors and dashed arrows indicate multiple reaction steps. Metabolite abbreviations used but not provided in the text are: glc-D, D-glucose; g6p, glucose-6-phosphate; 6pgc, 6-phospho-gluconate; ru5p-D, D-ribulose 5-phosphate; r5p, ribose-5-phosphate; e4p, erythrose-4-phosphate; f6p, fructose-6-phosphate; fru, D-fructose; g3p, glyceraldehyde 3-phosphate; 13dpg, 3-phospho-D-glycerol phosphate; 3pg, 3-phospho-glycerate; 2pg, 2-phospho-glycerate; pep, phosphoenolpyruvate; pyr, pyruvate; accoa, acetyl-CoA.

FOCAL can also create more complex coupling conditions, where not just one but multiple reactions are coupled to biomass production. One such potential application of this is to design strains that co-utilize multiple substrates in order to overcome difficulties associated with diauxic growth and to speed up fermentation. Using this approach, a strain was proposed using the iJO1366 model for *E. coli *that is incapable of growth unless the cell concurrently consumes both glucose and xylose (Figure 6). This mutant has defects in both the pentose phosphate and glycolysis pathways, making it incapable of producing NAD/NADP and membrane lipids unless both glucose and xylose are consumed (see Table S3 in Additional file 2 for a list of biomass components that cannot be made from individual sugars). The uptake of xylose and glucose concurrently allows the cell to produce dihydroxyacetone phosphate and glycerol-3-phosphate, which are used to produce NAD(P) and phospholipids. Such a mutant could be adaptively evolved to efficiently co-utilize both glucose and xylose under anaerobic conditions.

A major distinction between this particular FOCAL designed mutant and others designed using elementary modes [30] is that we can consider genome-scale networks and can enforce stricter co-utilization requirements. Unlike previous designs that can utilize either glucose or xylose for growth and ethanol production [30], our algorithm identified a mutant where it is mandatory to use both glucose and xylose in order to grow, creating a strong selection for co-utilization in adaptive evolutionary experiments. Evolved mutants could improve lignocellulose conversion and avoid the added complications of developing and maintaining a co-culture system [31]. By evolving co-utilizing mutants, progress could be made towards more efficient strains for production of biofuels from lignocellulosic biomass.

## Conclusions

FOCAL is capable of proposing experimental conditions (mutants and media composition) that will force coupling between a chosen flux of interest and a measurable flux (for example, cellular growth). As a result, FOCAL can design experiments to assess the accuracy and usage of metabolic reactions and their associated genes. FOCAL has numerous applications, including validating network elements, discovering new GPR associations and designing strains with unique and complex phenotypes. In addition, FOCAL coupling conditions could be used to select for improved enzyme activities since selection for improved growth would require improved flux through these reactions. Future work will look to reduce the total number of experiments needed to probe entire networks (by considering alternative solutions) and incorporate more advanced modeling components such as regulatory information to improve strategies proposed by the forced coupling algorithm.

## Materials and methods

### Forced coupling algorithm

**Figure 7:**
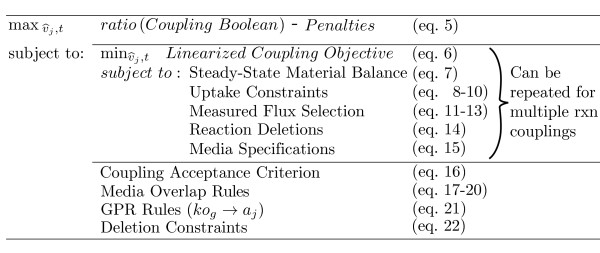
**Overview of the forced coupling algorithm (FOCAL)**.

FOCAL is a mixed-integer linear program (MILP) that works to propose media conditions and gene deletions such that a chosen flux (for example, fumarase) becomes coupled with another measurable flux (for example, cellular growth), meaning that flux through the measured reaction requires flux through the chosen reaction. FOCAL (summarized in Figure 7) is a bi-level algorithm composed of an inner problem that forces a flux ratio of interest to take its minimum value subject to media changes and gene deletions enforced by the outer problem. The outer problem searches for media conditions and a minimal number of deletions such that the minimum flux ratio is positive, ensuring that coupling occurs between a measurable flux (from a user specified set, *Coupling*) and the chosen reaction (that is, v_measured _→ v_chosen_). Since FOCAL calculates non-linear flux ratios, the problem must first be linearized in order to solve the problem as a MILP. Therefore, the non-linear problem is transformed to its linear form as described previously [14], in this case by normalizing flux through each reaction (j), including the chosen flux, by the measured flux:

(1) $$\frac{v_{chosen}}{v_{measured}}=v_{chosen}.t={\hat{v}}_{chosen}$$

(2) $$t=\frac{1}{v_{measured}},{\hat{v}}_{j}=v_{j}\cdot t$$

For this transformation to be valid, all fluxes must be non-negative; thus, each reversible reaction was decomposed into a forward and reverse reaction, and the resultant fluxes transformed as above:

(3) $${\hat{v}}_{j}={\hat{v}}_{j,for}-{\hat{v}}_{j,rev,}\forall j\in R$$

(4) $${\hat{v}}_{j,rev}=0,\forall j\notin R_{reversible}$$

where R is the set of all reactions, and R_reversible _refers to the subset of all reversible reactions. FOCAL is formulated using the Equations 5 to 22 listed below.

### Outer objective

(5) $$\text{max}\left(r_{obj}\left({{▵}_{ma}}_{x}+1\right)-1\right)-\alpha \;\Sigma_{g}\left(1-ko_{g}\right)-\beta \;\Sigma_{j}m_{j,additional}$$

### Inner objective

(6) $$\text{min}\;{\hat{v}}_{chosen,for}+{\hat{v}}_{chosen,rev}$$

#### Steady-state material balance

(7) Σ j ∈(Unblocked)Sij(v ^j,for-v ^j,rev)=0,∀i∈M

#### Uptake constraints

(8) $${\hat{v}}_{j,rev\;}\;\leq t\cdot {v_{j}}^{MaxUptake},\;\forall j\in \;Exch$$

(9) $${\hat{v}}_{j,for},{\hat{v}}_{j,rev},t\geq 0$$

(10) $${\hat{v}}_{j,rev}=0,\forall j\notin \;R_{reversible}$$

#### Select measured flux

(11) $${\hat{v}}_{j,for}=1,\;\;\;\;\;\;if\;c_{j,for}=1,\;\;\;\forall j\in Coupling$$

(12) $${\hat{v}}_{j,rev}=1,\;\;\;\;if\;c_{j,rev}=1,\;\;\forall j\in \;Coupling$$

(13) $$\Sigma \underset{Coupling}{j\;\in }\;\;c_{j,for}+c_{j,rev}=\;1$$

#### Reaction deletions

(14) $${\hat{v}}_{j,for},{\hat{v}}_{j,rev}=0,\;if\;a_{j}=0,\;\forall j\;\in \;R$$

#### Media specifications

(15) $${\hat{v}}_{x,rev}=0,if\;h_{x}=\;0,\;\forall x\;\in \;Exch\backslash Minimal$$

#### Coupling acceptance criterion

(16) $$\left({\hat{v}}_{chosen,for}+\;{\hat{v}}_{chosen,rev}\right)\;+\;\left(1\;-\;\in \right)\geq \;r_{obj}$$

#### Media overlap rules

(17) $$\underset{k\;\in \;K}{\sum }m_{x,k}\cdot media_{x,k}\;\geq \;h_{x},\;\;\;\;\;\;\forall x\;\in \;Exch$$

(18) $$m_{x,k}\leq h_{x},\;\;\;\forall k\;\in \;K,\forall x\;\;\in \;\;Exch$$

(19) $$\Sigma_{k\;\in \;K}m_{x,k\;}\cdot medial_{x,k}\;\leq \;1,\;\forall x\;\in \;Exch$$

#### Media uptake rules

(20) $$\Sigma \underset{Exch}{x\;\in }\;m_{x,k}\cdot media_{x,k}\;\leq \;\text{max}Media_{k},\;\forall k\;\in \;k$$

#### GPR rules

(21) $$a_{j}=f\left(ko_{g}\right)$$

#### Deletion constraints

(22) $$\Delta_{min}\leq \sum_{g\in G}\left(1-ko_{g}\right)\leq \Delta_{max}$$

### Inner primal problem

The inner problem (Equations 6 to 15) minimizes the ratio of the two fluxes for both chosen reaction directions such that if no coupling exists the inner objective is zero. This effectively amounts to solving the flux coupling framework problem proposed by Burgard *et al. *[14] to determine flux coupling, except, for their purposes, Burgard *et al. *also considered maximizing this objective. The transformed fluxes in the inner problem are subject to standard steady-state mass balance constraints (Equation 7), which ensure that there is no net production or consumption for the set of all metabolites, M. Equations 8 to 10 are identical to those reported previously [14] to constrain substrate uptake and the linearization variable, *t*. Here, *Exch *is the set of all exchange reactions, and $v_{j}^{MaxUptake^{\neg }}$ is the maximal substrate uptake flux for that exchange (see Additional file 1 for values used). Equations 11 to 13 are used to select the measured flux that will be coupled with the chosen flux of interest. The binary indicator variables, *c_j,for _*and *c_j,rev_*, are used to determine which flux, from a specified set of measurable fluxes (*Coupling*), *v_chosen _*is being coupled with. Any deleted reactions (as determined based on GPR rules, described below), indicated by binary variable, *a_j_*, have their flux set to 0 in both the forward and reverse directions using Equation 14. All conditional constraints (Equations 11, 12, 14 and 15), were implemented using GAMS (GAMS Development Corporation, Washington, DC, USA) indicator constraints.

To allow changes in minimal media conditions, four sets of media components were defined, each set containing exchange reactions used to import metabolites as sources of carbon, nitrogen, electron acceptors, or additional nutrients. Each media component set was specific for individual models. If experimental information was not available, FBA [6] was used to predict whether the microbe could use metabolites as a carbon, nitrogen or electron acceptor source or as an additional media component (media sets defined in Additional file 1). Note these media component sets are largely a bookkeeping mechanism for the user; thus, while a component may be categorized as a particular nutrient source, this does not mean that the organism will use this metabolite strictly for this purpose (for example, putrescine may be selected as nitrogen source, but may also be used as a carbon source in the model). Equation 15 allows FOCAL to define the minimal media to be tested whilst removing all unselected substrate exchanges. Here, *Minimal *is the set of exchange fluxes that are essential for cellular growth irrespective of the carbon, nitrogen or electron acceptor selected (for example, water, protons, essential salts, ions, phosphate, and sulfur sources).

### Outer problem

In the outer problem, a binary indicator variable, *r_obj_*, is used to determine whether the desired coupling criterion has been satisfied (that is, ${\hat{v}}_{chosen,for}+{\hat{v}}_{chosen,rev}>\in$) using the acceptance criteria constraint (Equation 16). For the *E. coli *and *B. subtillis *models, ε was set to 10^-5 ^while for *P. putida *this was increased to 10^-4 ^due to scaling differences between the models. To allow FOCAL to design media conditions, the media selection rules (Equation 17 to 19) were implemented as part of FOCAL's outer problem in which *m_x,k _*is a binary indicator variable used to select a metabolite exchange, *x*, from one of the created media component sets, while *media_x,k _*is a binary matrix indicating whether metabolite exchange, *x*, belongs to the media component type, k. K is the set of four media component types (carbon source, nitrogen source, electron acceptor, and additional nutrients), and *h_x _*is a binary variable used to control the media composition and uptake rates in the inner problem (Equation 15). An optional constraint (Equation 19) prevents a given metabolite exchange from being selected as more than one media component type. Equation 20 also limits the total number of metabolite exchanges that can be used for each media component type. The parameter *maxMedia_k _*was set to one, except for the co-utilization case, where it was set to two to enable use of two carbon sources.

FOCAL is subject to GPR deletion rules (Equation 21), which were implemented as described previously [32]. Such rules use a series of binary variables to map gene deletions (ko_g _= 0) to associated reaction deletions (a_j _= 0). Limits on the maximum and minimum number of gene deletions were also imposed considering the set of genes in the model, G (Equation 22), using parameters Δ_max _and Δ_min_. For these studies, Δ_max _and Δ_min _were normally set to 5 and 0, respectively. In FOCAL's outer objective function (Equation 5), *α *and *β *are positive real numbers used to penalize the use of gene deletions and metabolites from the additional nutrient set. For this study, values of *α *= 1.0 and *β *= 0.25 were used so that adding additional nutrients would be favored over creating extra deletions, which take more time experimentally. FOCAL is not very sensitive to the penalty values, so these values can be changed to modify the type of proposed experiments as long as the maximum possible combined penalties do not exceed the increase in the objective resulting from the desired coupling. To solve the bi-level problem using available MILP solvers, the inner problem is rewritten using duality such that the primal and its dual are solved simultaneously and their objectives set equal to one another. This guarantees that the inner problem objective is met prior to maximizing the outer objective [33]. Complete formulation of FOCAL as a single-level MILP is provided in Figure S6 in Additional file 2. An implementation of FOCAL in GAMS (GAMS Development Corporation) for the example network shown in Figure 2 is provided in Additional file 3.

### Evaluation of different networks

Using FOCAL, coupling conditions were proposed for reactions within the genome-scale models of *E. coli *[20,21], *B. subtilis *[22], and *P. putida *[23]. Given the increased size of the network and complexity of GPRs in iJO1366 (Table S4 in Additional file 2), we first reduced the number of gene deletion decision variables for this model by excluding subunits and isozymes as described by Hamilton and Reed [34]. We also replaced the Nuo and Ndh reactions in iJO1366 with average reactions since the flux through these reactions was constrained to be equal [21]. Additionally, we removed the wild-type biomass from the network and based all coupling off of the core biomass equation.

For simplicity, a reaction flux was considered coupled to a measurable flux if a media and gene knockout strategy could be generated for either its forward or reverse component (Equations 6 and 16). To improve run-time performance, the set of possible measurable fluxes (that is, those in *Coupling*) that a chosen reaction could be coupled with initially only contained the biomass flux. For *E. coli *reactions for which FOCAL could not initially find a coupling condition, FOCAL was re-run using an expanded *Coupling *set that included ethanol, formate, and succinate secretion in addition to the biomass, since these metabolites are common anaerobic by-products and can be easily measured.

CPLEX can take a significant amount of time to find and prove that a solution is the global minimum. Since we were mainly interested in finding FOCAL solutions for all reactions in the genome-scale networks and not necessarily finding the global minimum, we limited the time FOCAL could spend searching for a better solution; however, this is not required if one desires to obtain a global solution. Using a CPLEX option (tilim), the algorithm was allowed only 3 hours to find a solution for any given reaction coupling problem. To further reduce the time spent solving for an optimal solution, once a feasible solution to the coupling problem was discovered, the algorithm was only allowed an additional 10 minutes to search for a better solution using the GAMS BCH facility. To minimize the number of different minimal media conditions proposed and to prune simple coupling problems, a reduced set of metabolite exchange reactions composed of glucose, ammonium, and oxygen exchanges as well as the entire additional nutrient set was used for the initial 10 minutes of solution time. If no solution was found within this time period, then a more exhaustive search was performed using all elements within the various media component sets for the remainder of the allotted 3 hours. This amount of time is comparable to other bi-level MILP methods given the number of decision variables involved. Further improvements in run-time performance may be possible by constraining the dual variables [35] or eliminating gene deletion decision variables for reactions that are coupled to other reactions under all media conditions [14]. Media component sets for the different models and run-time statistics are provided in Additional file 1 and Table S5 in Additional file 2 respectively.

### Discovery of alternative solutions

FOCAL will initially only propose a single coupling condition that best maximizes the objective. Under certain circumstances, alternative solutions may exist and can be found by adding integer cut constraints that make prior FOCAL solutions infeasible:

(23) $$\underset{g\in OldKO}{\sum }\left(1-ko_{g}\right)+\underset{j\in OldMedia}{\sum }\left(h_{j}\right)\leq \left|OldKO\right|+\left|OldMedia\right|-1$$

where *OldKO *is the set of genes deleted in a past solution and *OldMedia *is the set of media components proposed in that same solution. Such a cut prevents FOCAL from proposing a solution that is identical to or a superset of a previous solution. Additionally, one can omit the knockout or media component of the integer cut depending on the type of alternative solutions one is interested in obtaining.

### Strains

*E. coli *strains from the Keio collection [24], specifically *uxaA*::kan, *uxaB*::kan, *uxaC*::kan, *uxuA*::kan, *uxuB*::kan, *yeiQ*::kan, and *E. coli *K-12 BW25113, were used in FOCAL designed experiments. Additionally, three double mutants (*ΔuxuB*::kan *ΔuxaB, ΔyeiQ*::kan *ΔuxaB*, and *ΔyeiQ*::kan *ΔuxuB*) and a triple mutant (*ΔyeiQ*::kan *ΔuxuB ΔuxaB*) were generated using sequential removal of the kan gene using FLP recombinase [36] and P1 transduction [37] followed with selection for kanomycin resistance.

### Growth phenotype plate experiments

All strains were grown in triplicate at 37°C in a Tecan Infinite 200 microplate reader (Tecan Group Ltd, Switzerland) using 96-well plates. OD measurements were taken at 600 nm every 15 minutes with linear shaking (830 seconds, 4.5 mm). Tecan OD measurements were converted to an equivalent OD_600 _value in a Biomate spectrophotometer with a 1 cm path length (see [10] for conversion factors used). All strains were pre-cultured for approximately 24 hours in M9 medium supplemented with 2 g/L glucose and subsequently washed twice with M9 minimal media containing no carbon source to remove any residual glucose. Cells were then resuspended in different media - M9 + 2 g/L D-galacturonate or M9 + 2 g/L D-glucuronate - such that the starting OD_600 _measurement was approximately 0.05 and then grown in the Tecan plate reader.

## Abbreviations

FBA: flux balance analysis; FOCAL: forced coupling algorithm; GEM: genome-scale model; GPR: gene-protein-reaction; MCS: minimal cut set; MILP: mixed-integer linear program; OD: optical density.

## Authors' contributions

CJT developed the algorithm, processing scripts, and databases, performed all the simulations, conducted all the experiments and composed all figures. CJT and JLR conceived of and designed the algorithm and experiments, analyzed results and wrote the paper. All authors read and approved the final manuscript.

## Supplementary Material

Additional file 1 **List of the maximum uptake rates and media components for the three genome-scale models**. Click here for file

Additional file 2 **Supplementary material, including additional algorithm details, supplementary tables, and supplementary figures**. Click here for file

Additional file 3 **An implementation of FOCAL for the example network shown in Figure 2**. This file can be run by GAMS (GAMS Development Corporation, Washington, DC), which can be freely downloaded at [38]. Click here for file
