# Supplementary material for: FOCAL: an experimental design tool for systematizing metabolic discoveries and model development
Source: Genome Biol. 2012 Dec 13;13(12):R116. doi: 10.1186/gb-2012-13-12-r116 (PMC4056367; doi:10.1186/gb-2012-13-12-r116)
Supplement: Additional file 2 — Supplementary material, including additional algorithm details, supplementary tables, and supplementary figures. [file gb-2012-13-12-r116-S2.PDF]

Table of Contents

	Page
**1. Supplemental Methods**	
1.1. Calculating Shortest Path Between Reactions	2
1.2. Determination of Essential and Blocked Reactions and Genes	2
1.3. Multi-Reaction Coupling in FOCAL for Co-Utilization	3
1.4. Biomass Components Produced by Substrates	3
1.5. Determination of Gene-Reaction Clusters	4
1.6. Determination of Network Cycles	4
**2. Supplemental Figures and Tables**	
Figure S1. Media Component Selection for *E. coli* FOCAL Experiments	4
Figure S2. Additional Nutrient Selection for *E. coli* and *B. subtilis* Experiments	7
Figure S3. Breakdown for *E.coli* (iJO1366) Coupled and Uncoupled Reactions	7
Figure S4. Examples of Reaction Structures that Cannot Be Coupled	8
Figure S5. Growth Phenotypes of Mutants on Glucuronate and Galacturonate	9
Figure S6. Single-Level MILP Formulation of the FOCAL algorithm	10
Table S1. Most Common Deletions for Each Genome-Scale Network	11
Table S2. Reaction Coupling Conditions for Fluxes with No Associated GPRs	11
Table S3. Biomass Components Produced by Glucose or Xylose	12
Table S4. Comparison of GPR Complexity between iJR904 and iJO1366	12
Table S5. Average Time Statistics Across the Various Networks	12
**3. References**	13

1. Methods

1.1. Calculating Shortest Path Between Reactions

For all *E. coli* gene deletions associated with a single reaction, the shortest distance between the associated reaction and the reaction to be coupled to biomass (or some other measurable flux) was calculated. This was accomplished by removing currency metabolites (protons, water, nad, nadp, amp, adp, atp, and phosphate) from the metabolic network and subsequently creating a new flux centric network. In this modified network, reactions are nodes, and edges are mappings from a reaction's products to a reaction that uses those metabolites as reactants. All edges are assigned a weight of one and distances are calculated for both a directed and undirected version of the network. In the directed case, the shortest distance is determined by solving the following network balance problem:

$$\min \sum_{\substack{(i,j) \\ \in A(i,j)}} p_{ij} \tag{1}$$

$$\left(\sum_{j \in A(j,i)} p_{ji} - \sum_{j \in A(i,j)} p_{ij} \right) = (Sink_i - Source_i) * (1 - 2s), \quad \forall i \in R \tag{2}$$

$$s \in \{0,1\} \tag{3}$$

Where A_{ij} is the set of all network arcs (i.e., a set of connections between reaction node i which shares a metabolite with reaction node j), p_{ij} is a flow variable which indicates flow over an edge, $Sink_i$ indicates the deleted reaction node, $Source_i$ indicates the coupled to reaction node, R is the set of all reaction nodes and s is a binary variable used to swap source and sink directions.

For the undirected case, all reactions are treated as if they are reversible. This is accomplished by writing the problem as:

$$\min \sum_{\substack{(i,j) \\ \in A(i,j)}} (p_{ij} + rp_{ij}) \tag{4}$$

$$\left(\sum_{j \in A(j,i)} p_{ji} - \sum_{j \in A(i,j)} p_{ij} \right) + \left(\sum_{j \in A(i,j)} rp_{ji} - \sum_{j \in A(j,i)} rp_{ij} \right) = (Sink_i - Source_i), \quad \forall i \in R \tag{5}$$

Where all symbols are as defined above and rp_{ij} represents reverse flow through an edge. Since directionality is irrelevant under these circumstances, the use of binary variable, s, is unnecessary.

1.2. Determination of Essential and Blocked Reactions and Genes

To reduce the number of genes considered as possible deletions, blocked reactions were found as described by Burgard et al.[1] with one amendment. To prevent cyclic behavior with blocked reversible reactions, the reverse reaction is removed from the system before solving the blocked reaction LP. If a reaction is unable to sustain flux even with all possible exchange fluxes open, the reaction is added to the blocked set and genes associated exclusively with these reactions are excluded from consideration as possible deletions. Similarly, if a given gene deletion prevented the cell from growth with all exchange fluxes open, it was categorized as essential and removed from the list of potential deletions.

1.3. Multi-Reaction Coupling in FOCAL for Co-Utilization

To apply FOCAL to the problem of co-utilization of multiple substrates, multiple forced coupling problems are solved simultaneously, working to couple biomass to the uptake of all chemical species of interest. To account for these additional inner problems, the objective and problem are rewritten in the following form:

Multiple Problems Outer Objective:

$$\max \ (r'(\Delta_{max} + 1) - 1) - \alpha \sum_{g} (1 - ko_g) - \beta \sum_{j} m_{j,additional} \tag{6}$$

Where r' is a binary indicator variable used to determine if all the provided inner problems have been coupled. An example with two inner problems is shown below:

Inner Problems

$$\min \ \hat{v}_{chosen1,for,p1} + \hat{v}_{chosen1,rev,p1}$$
$$s.t. \ Forced\ Coupling\ Inner\ Constraints$$
$$\min \ \hat{v}_{chosen2,for,p2} + \hat{v}_{chosen2,rev,p2}$$
$$s.t. \ Forced\ Coupling\ Inner\ Constraints$$

General Forced Coupling Outer Constraints

$$Acceptance\ Criteria,\ \ r_1$$
$$Acceptance\ Criteria,\ r_2$$

Ratio Handling

$$(r_1 - 1) + (r_2 - 1) \leq (r' - 1) \tag{7}$$

$$r_1, r_2 \geq r' \tag{8}$$

OPTIONAL: Force Infeasibility or User Specified Deletions

$$r_1, r_2 \geq \epsilon \tag{9}$$

$$\hat{v}_{j,for}, \hat{v}_{j,rev} = 0, \ \ if\ User\_Deletions_j = 0, \ \ \forall j \in R \tag{10}$$

For the xylose-glucose co-utilization experiments, the Δ_{max} constraint was removed, and the media sets were modified so that the minimal media contained exclusively xylose, glucose, ammonium and essential metabolites. Forcing infeasibility is a potentially useful constraint when the solver struggles to find a solution in a timely manner. While only two inner problems are shown here, the algorithm can be expanded to handle any number of flux couplings.

1.4. Substrate Contribution to Biomass

Sink reactions (e.g. metabolite $\rightarrow$) were added to iJR904 for each metabolite that is a reactant in the biomass reaction. A MILP was then constructed of the following form:

$$\max \sum_{j \in S} Component_j, \qquad \forall j \in S \tag{11}$$

$$\sum_{j \in R} S_{ij} v_j = 0, \qquad \forall i \in M \tag{12}$$

$$LB_j \leq v_j \leq UB_j, \qquad \forall j \in R \tag{13}$$

$$v_j \ \geq \ \epsilon \cdot Component_j, \qquad \forall j \in S \tag{14}$$

$$Component_j \ \in \ \{0,1\} \tag{15}$$

Here M is the set of all metabolites, R is the set of all reactions, S is the subset of added sink reactions, and LB_j and UB_j are the flux lower and upper bounds respectively for a given reaction j. ε is a small number (~10^{-3}) that is used to determine if there is flux through v_j. For a given mutant, GPR rules are used to fix deleted reactions to a zero flux. The above problem was solved for each individual substrate to be co-utilized to determine what biomass components each substrate could produce individually (see **Table S3** for results).

<u>1.5. Determination of Gene-Reaction Cluster</u>

In order to more easily evaluate the interrelationship between genes and their associated reactions, we developed the following method to determine distinct gene-reactions clusters from GPR relationships. To accomplish this, one starts by selecting a gene that is not presently associated with a cluster and adding it to a new set, *GeneMembers*. This gene is then mapped to all associated reactions, which are added to a new set, *RxnMembers*. Subsequently, all of these reactions are mapped to their associated genes, which are added to the *GeneMembers* set. The process of adding to the two sets is repeated iteratively until no more genes or reactions are added to the sets. At this point, the grouping of the *RxnMembers* and *GeneMembers* sets is stored as a unique and distinct gene-reaction cluster. This process is repeated until all genes possessing a GPR belong to a cluster.

<u>1.6. Determination of Network Cycles</u>

The following integer program (IP) can be used to determine various cycles within a model where one metabolite can be traced backed to itself through a series of reactions. This is accomplished by effectively performing a breadth-first search by mapping substrates to their associated reactions and then to associated products. This entire step (substrate to reaction to product) is considered an increase in depth (*d)* of 1, while two metabolites connected by two reactions would have a depth of 2. The user picks a metabolite of interest, *MoI*, from which they desire to begin and end a cycle. A cycle is found mathematically as follows:

$$\max \ \alpha * \sum_{i \in MoI} isEnd_i - \sum_{(j,d) \in R,D} Rxn_{j,d} + RxnRev_{j,d} \tag{16}$$

$$\sum_{\substack{i \in \\ Metab2Rxn_{i,j}}} Switch_{i,d} \geq Rxn_{j,d}\,, \qquad \forall j \in R, \forall d \in D \backslash \{|D|-2\} \tag{17}$$

$$\sum_{\substack{i \in \\ Metab2RxnRev_{i,j}}} Switch_{i,d} \geq RxnRev_{j,d}\,, \qquad \forall j \in R,, \forall d \in D \backslash \{|D|-2\} \tag{18}$$

$$\sum_{\substack{j \in \\ Rxn2Metab_{j,i}}} Rxn_{j,d} + \sum_{\substack{j \in \\ Rxn2MetabRev_{j,i}}} RxnRev_{j,d} \geq Switch_{i,d+1}, \qquad \forall i \in M, \forall d \in D \backslash \{|D|-2\} \tag{19}$$

$$\sum_{d \in D} (Rxn_{j,d} + RxnRev_{j,d}) + RxnBlock_j \leq 1 , \qquad \forall j \in R \tag{20}$$

$$\sum_{d \in D \setminus \{0\}} Switch_{i,d} \geq isEnd_i , \qquad \forall i \in MoI \tag{21}$$

$$Switch_{i,0} = 1 , \qquad \forall i \in MoI \tag{22}$$

$$Switch_{i,0} = 0 , \qquad \forall i \notin MoI \tag{23}$$

$$Switch_{i,d}, Rxn_{j,d}, RxnRev_{j,d}, isEnd_i \in \{0,1\} \tag{24}$$

Here D is the set of indices indicating the depth level (i.e., D = {0,1…,|D|-1}), R is the set of all reactions, $Metab2Rxn_{i,j}$ is the mapping of all substrates - excluding currency metabolites – to their associated reactions (forward direction), $Metab2RxnRev_{i,j}$ is an identical mapping except for reactions running in reverse, $Rxn2Metab_{j,i}$ is a mapping of all reactions (forward direction) to their associated products, similarly $Rxn2MetabRev_{j,i}$ an identical mapping but for reactions running in reverse, α is an arbitrary number >> 1 used to reward the algorithm for completing a cycle. For our purposes, we used an $\alpha = 1000$. $isEnd_i$ is a binary variable indicating a cycle has been completed, $Switch_{i,d}$ is a binary variable that indicates whether a metabolite, *i*, can and is produced in the cycle at a depth, *d*, and $Rxn_{j,d}$ and $RxnRev_{j,d}$ are binary variables indicating whether a forward or reverse reaction, *j*, respectively, is used at depth, *d*. $RxnBlock_j$ is a user defined vector of 0s and 1s used to remove any blocked reaction, *j*, from the network, where 1 indicates a reaction is blocked. Alternative cycles can be determined by applying an integer cut on the reactions used to create a cycle. With slight adjustments, this algorithm can also potentially be used to determine a path between a given metabolite and biomass components which may be helpful in determining similar reactions in terms of their end contribution to biomass.

2. Supplemental Figures and Tables

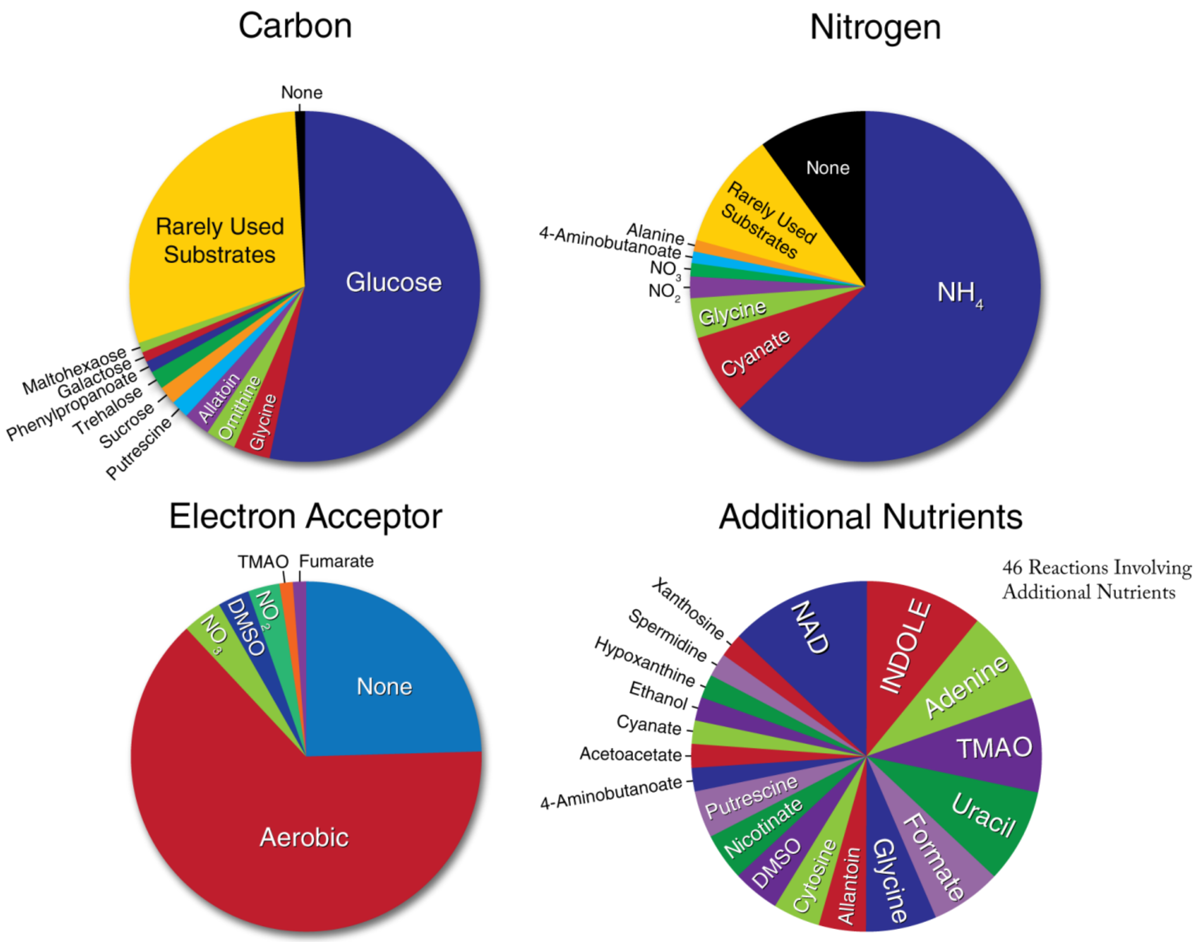

Figure S1. Media Component Selection for *E. coli* (iJR904) FOCAL Experiments. 'Rarely Used Substrates' contains various metabolites that were proposed less than six times while 'none' indicates that a metabolite from another category is being used as a source of multiple essential elements (e.g., putrescine is being used as both a carbon and nitrogen source). For additional nutrients, only 46 chosen reactions required a metabolite from this set, and the thus chart does not represent distribution for all coupling conditions proposed.

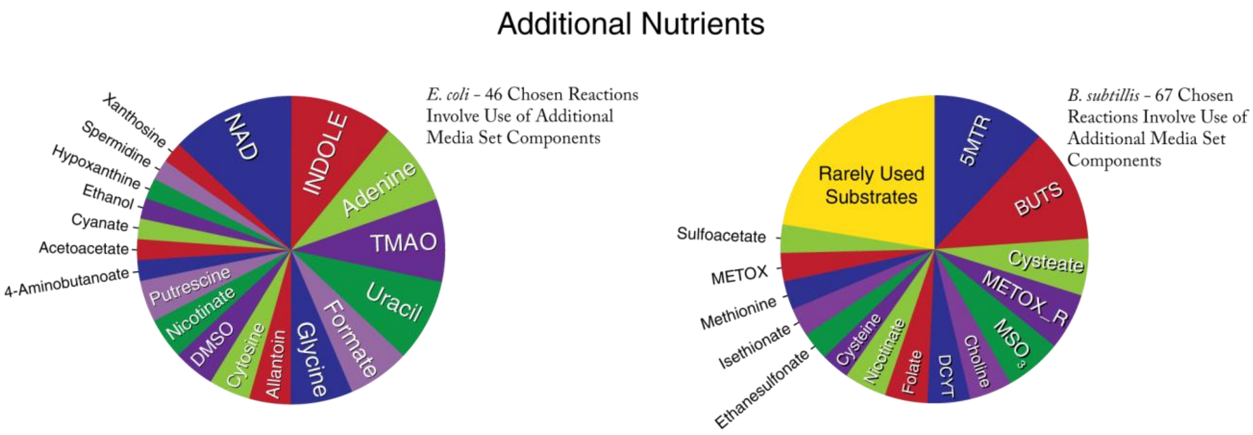

Figure S2. Additional Nutrient Selection for *E. coli* (iJR904) and *B. subtilis* Experiments. 'Rarely Used Substrates' is composed of various metabolites that were selected less than 2 times. Only 46 and 67 chosen reactions for *E. coli* and *B. subtillis* respectively required a metabolite from this set, and thus the chart does not represent distribution for all coupling conditions proposed. The following abbreviations were used: 5MTR, 5-Methylthio-D-ribose; BUTS, butanesulfonate; METOX, L-Methionine S-oxide; METOX_R, L-methionine R-oxide; MSO_3, Methanesulfonate; DCYT, Deoxycytidine;

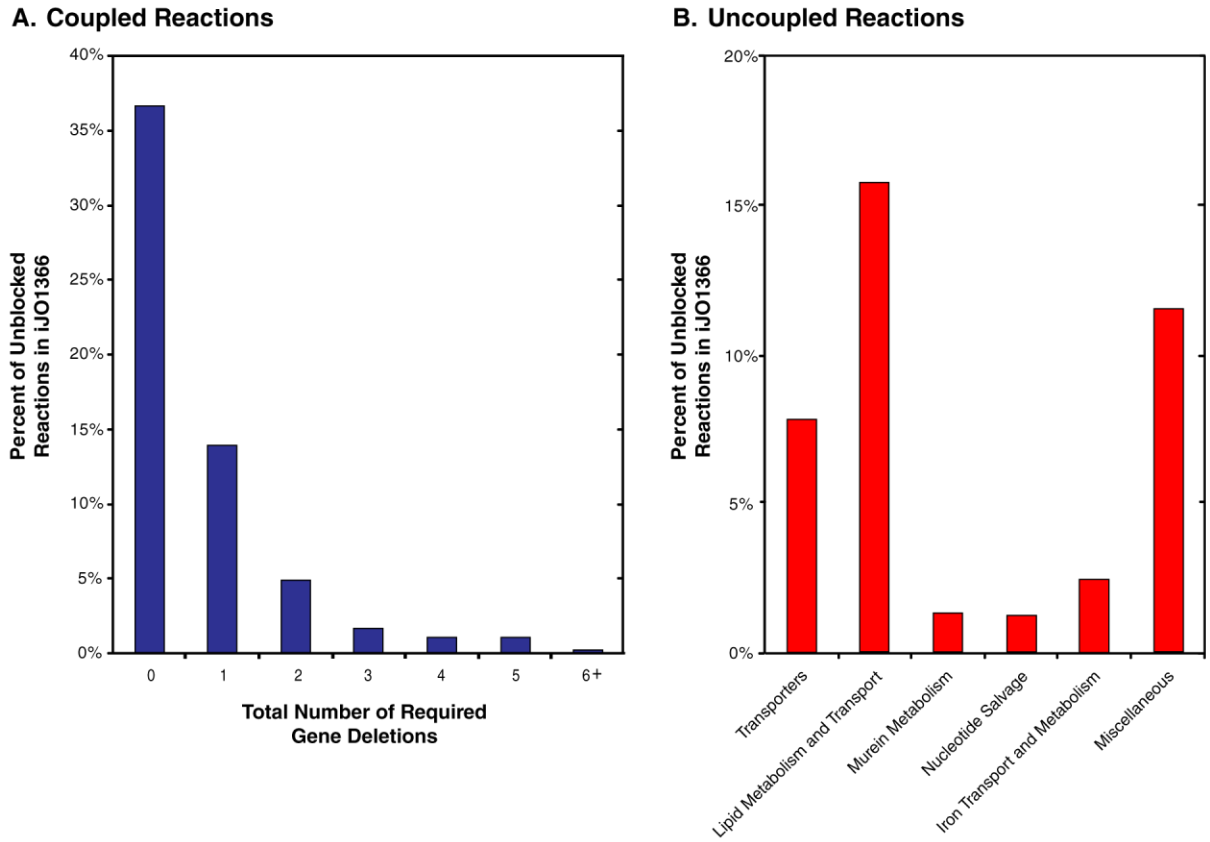

Figure S3. Breakdown for *E. coli* (iJO1366) Coupled and Uncoupled Reactions. A.) A complete picture of iJO1366 gene deletion proposals including isozyme deletions. B.) A breakdown of uncoupled reactions by their metabolic function. The category miscellaneous contains groupings of reactions that were not large enough to warrant their own category as well as various reactions that did not appear to fit into a given metabolic function.

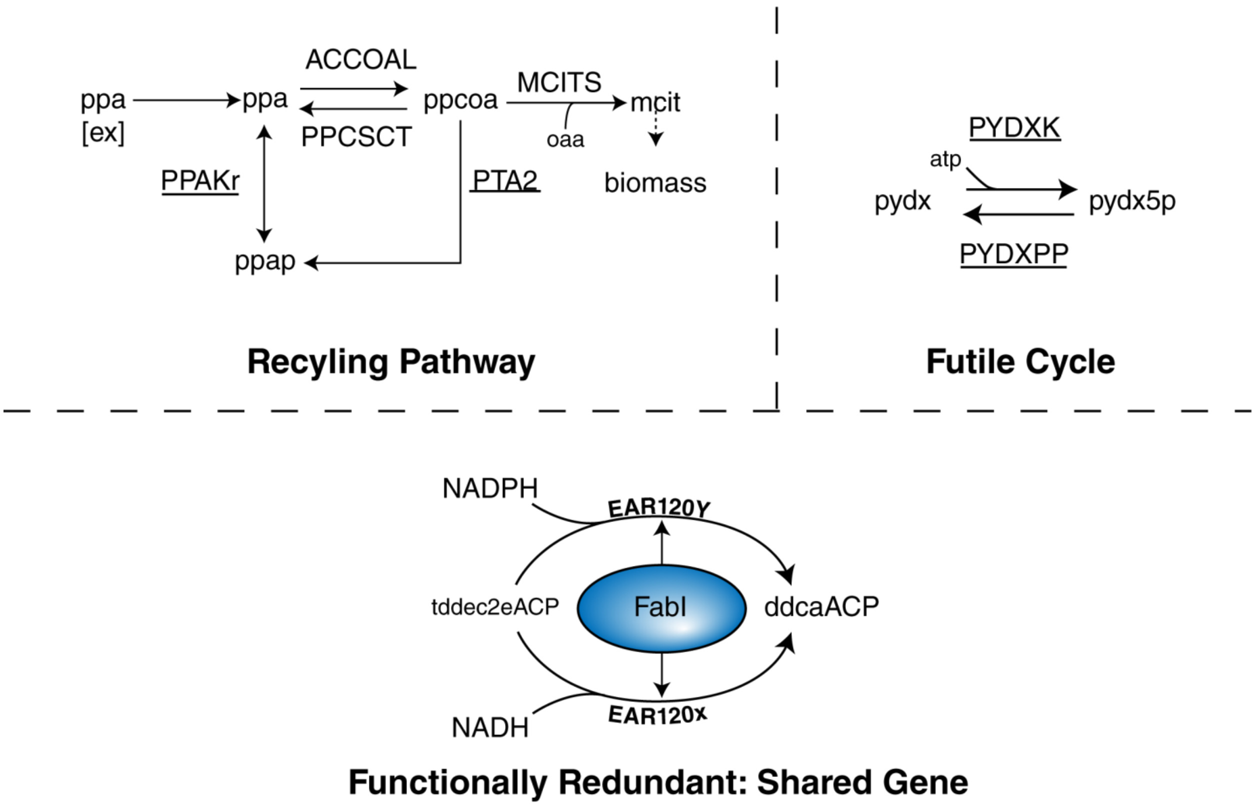

Figure S4. Examples of Reactions that Cannot be Coupled. Some common reasons why reactions cannot be coupled to biomass. In the recycling pathway, the underlined reactions (PTA2 and PPAKr) cannot be coupled to biomass since they are not needed to convert propionate (ppa) into biomass. In the futile cycle example, the reactions PYDXK and PYDXPP must have the same flux values and the net effect is ATP hydrolysis, which is not necessary for biomass production. With functionally redundant reactions, the two reactions are catalyzed by the same gene product; consequently, removing one will also remove the other making coupling impossible. A similar situation occurs when one of the functionally redundant reactions is not associated with any genes or is associated with an essential gene.

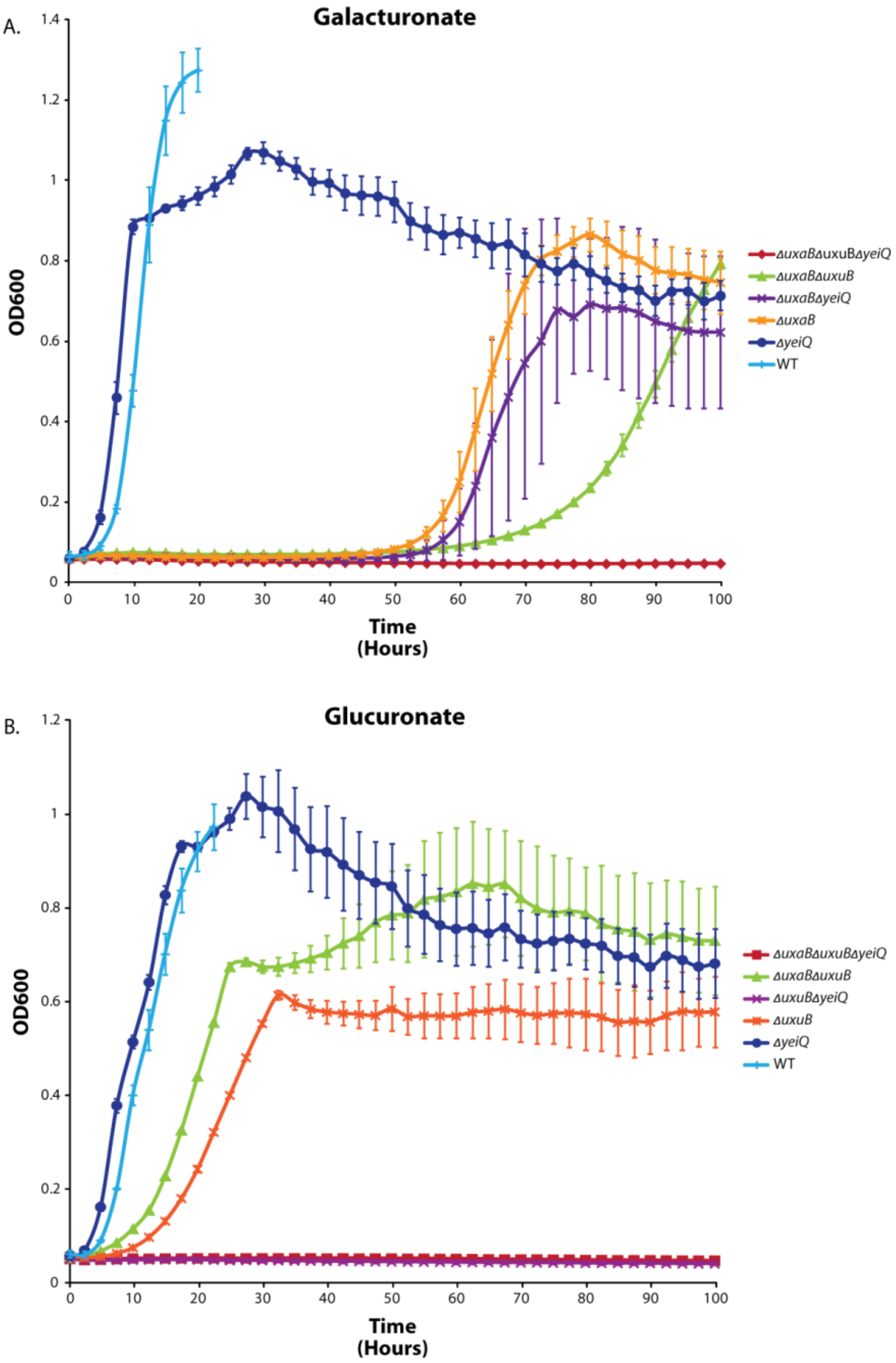

Figure S5. **Growth Phenotypes of Mutants on Glucuronate and Galacturonate**. The Δ*uxaB*Δ*yeiQ* mutant exhibited higher biological variability compared to the other mutants tested. In a separate experiment (not shown), only two out of three biological replicates grew after 4 days and all runs had large standard deviations between triplicates.

$$\max_{\hat{v}_j,t} \quad (r_{obj}(\Delta_{max}+1)-1) - \alpha \sum_g (1-ko_g) - \beta \sum_j m_{j,additional}$$

Primal Problem:

$$\sum_{j \in Unblocked} S_{ij}(\hat{v}_{j,for} - \hat{v}_{j,rev}) = 0, \quad \forall i \in M$$

$$\hat{v}_{j,rev} \leq -t * v_{uptake}^{max}, \quad \forall j \in Exch$$

$$\hat{v}_{j,dir} = 0, \quad \forall dir, \forall j \mid a_j = 0$$

$$\hat{v}_{j,rev} = 0, \quad \forall j \in Overlap \mid h_j = 0$$

$$\hat{v}_{j,rev} = 0, \quad \forall j \in (Exch \cap Overlap \cap Minimal) \mid h_j = 0$$

$$\hat{v}_{j,dir} = 1, \quad if\ c_{j,dir} = 1, \forall (j,dir) \in Coupling$$

$$\sum_{(j,dir) \in Coupling} c_{j,dir} = 1,$$

$$\hat{v}_{j,for}, \hat{v}_{j,rev}, t \geq 0$$

Dual Problem:

$$\sum_{i \in Unblocked_{j,for}} S_{ij} u_i^{mat} + u_j^2 = d_{j,for}, \quad \forall j \in R$$

$$\sum_{i \in Unblocked_{j,for}} S_{ij} u_i^{mat} + u_j^2 + u_j^{couple} = d_{j,for}, \quad \forall (j,for) | Coupling$$

$$\sum_{i \in Unblocked_{j,rev}} -S_{ij} u_i^{mat} + u_j^3 = d_{j,rev}, \quad \forall j \in R$$

$$\sum_{i \in Unblocked_{j,rev}} -S_{ij} u_i^{mat} + u_j^3 + u_j^{couplerev} = d_{j,rev}, \quad \forall (j,rev) \,| Coupling$$

$$\sum_{j \in Exch | Unblocked_{j,rev}} v_{uptake}^{max} u_j^t \leq 0,$$

$$u_j^2 = 0, \quad if\ a_j = 1, \forall j \in \pi_j\,(GPR_{j,n,g})$$

$$u_j^2 = 0, \quad \forall j \notin \pi_j\,(GPR_{j,n,g})$$

$$u_j^{couple} = 0, \quad if\ c_{j,for} = 0, \forall (j,for) \in Couple$$

$$u_j^{couplerev} = 0, \quad if\ c_{j,rev} = 0, \forall (j,rev) \in Couple$$

$$u_j^3 = 0, \quad if\ a_j = 1, \forall j \in \pi_j\,(GPR_{j,n,g})$$

$$u_j^3 = 0, \quad if\ h_j = 1, \forall j \in Overlap$$

$$u_j^3 = 0, \quad \forall j \in Minimal$$

$$u_j^3 = 0, \quad \forall j \notin ((\pi_j\,(GPR_{j,n,g})) \cup Exch)$$

Primal-Dual Equality:

$$\sum_{(j,for) \in Couple} u_j^{couple} + \sum_{(j,rev) \in Couple} u_j^{couplerev} = \sum_{j \in Unblocked_{j,for}} d_{j,for} \hat{v}_{j,for} + \sum_{j \in Unblocked_{j,rev}} d_{j,rev} \hat{v}_{j,rev}$$

Algorithm Parameters:

$$\sum_{x \in Exch} m_{x,k} \cdot media_{x,k} \leq maxMedia_k \quad \forall k \in K$$

$$\Delta_{min} \leq \sum_{g \in G} (1 - ko_g) \leq \Delta_{max}$$

GPR Rules:

$$\sum_{g \in GPR_{j,n,g}} (ko_g - 1) \leq Isozyme_{j,n} - 1 \quad \forall (j,n) \in \pi_{j,n} GPR_{j,n,g}$$

$$Isozyme_{j,n} \in \{0,1\}$$

$$ko_g \geq Isozyme_{j,n} \quad \forall (j,n,g) \in GPR_{j,n,g}$$

$$a_j \leq \sum_{n \in \pi_{j,n} GPR_{j,n,g}} Isozyme_{j,n} \quad \forall j \in \pi_j\, GPR_{j,n,g}$$

$$a_j \leq Isozyme_{j,n} \quad \forall (j,n) \in \pi_{j,n} GPR_{j,n,g}$$

Overlap Rules:

$$\sum_{k \in K} m_{x,k} \cdot media_{x,k} \leq 1 \quad \forall x \in Overlap$$

$$\sum_{k \in K} m_{x,k} \cdot media_{x,k} \leq h_x \quad \forall x \in Overlap$$

$$m_{x,k} \leq h_x \quad \forall x \in Overlap, \forall k \in K$$

Acceptance Criteria:

$$r_{obj} \leq \sum_{j \in Unblocked_{j,for}} d_{j,for} \hat{v}_{j,for} + \sum_{j \in Unblocked_{j,rev}} d_{j,rev} \hat{v}_{j,rev} + (1 - \epsilon)$$

Figure S6. Single-Level MILP Formulation of the FOCAL algorithm. Items are defined as in the main methods section. The set *Overlap* is defined as the union of all media types contained in the $Media_{x,k}$ matrix.

Table S1. Most common gene deletions for three of the genome-scale networks.

***E. coli* (iJR904)**				***E. coli* (iJO1366)**				***B. subtillis***				***P. putida***			
**Gene**	**Locus**	**Associated Reactions**	**Freq**	**Gene**	**Locus**	**Associated Reactions**	**Freq**	**Gene**	**Locus**	**Associated Reactions**	**Freq**	**Gene**	**Locus**	**Associated Reactions**	**Freq**
b2779	eno	ENO	16	b1288	fabI	EAR(80-181)(x/y), EGMEACPR, EPMEACPR	34	BG13378	mtnZ	SADT1r	32	PP4193	sdhC	RR00219, RR04368	9
b0451	amtB	NH3t	15	b4301	sgcE	RPE	28	BG10942	ywlF	RPI	9	PP0736	murI	RR00126	9
b4232	fbp	FBP	13	b3956	ppc	PPC	22	BG11426	nrdI	RNDR(1-4)	9	PP4187	lpdG	IR03527, IR08963, RR00772, RR00772	8
b0004	thrC	4HTHRS, THRS	11	b0008	talB	TALA	19	BG11331	drm	PPM,PPM2	8	PP4840	cycA	RR08634, RR08706	5
b2406	xapB	ADNt2r, CYTDt2r, INSt2r, THMDt2r, URIt2r, XTSNt2r	10	b3916	pfkA	PFK,PFK_2,PFK_3	14	BG10722	rocD	ORNTA	8	PP1009	gap-1	RR00685	5
b4154	frdA	FRD2, FRD3	9	b0452	tesB	FACOAE(80-181)	14	BG12930	yfkN	NTD(1-10), 23CN2P(1-4), 3NUCLE(1-4)	8	PP3443	-	IR00683	5
b4238	nrdD	RNTR(1-4)	9	b1702	ppsA	PPS	11	BG10413	ywjH	TAL	7	PP1768	serC	RR02325, RR02629	5
b0337	codA	CSND	8	b4238	nrdD	RNTR(1-4)c2	9	BG11044	adeC	ADD	7	PP4617	-	IR02216, RR00708, RR03658, RR10392	5
b3739	atpI	ATPS4r	8	b0221	fadE	ACOAD(1-8)f	8	BG11738	ygjI	PGDHr	7	PP5419	atpB	RR08593	5
b0621	dcuC	FUMt2_3, SUCCt2b, SUCFUMt	8	b2551	glyA	ALATA_D2, ALATA_L2, GHMT2r, THFAT, THRA2i, THRAi	7	BG12546	yxkJ	MALt2, CITt6	6	PP3590	tyrB-2	IR05334, IR09746, RR00397, RR00432, RR02327, RR04241	4

Table S2. Reaction Coupling Conditions for Fluxes with No Associated GPRs in iJR904

Rxn Label	Chosen Reaction	Genes to Deleter	Carbon	Nitrogen	Acceptors	Additional Media
A5PISO	[c]ru5p-D <==> ara5p	-	Glucose	Ammonium	Oxygen	-
ABUTD	[c]4abutn + h2o + nad --> 4abut + (2) h + nadh	-	Acetaldehyde	Putrescine	Oxygen	-
ALATA_L	[c]akg + ala-L <==> glu-L + pyr	-	Glucose	Ammonium	Oxygen	-
BPNT	[c]h2o + pap --> amp + pi	-	Glucose	Ammonium	Oxygen	-
CMPN	[c]cmp + h2o --> csn + r5p	b0910 b2143 b2406	Glucose	Ammonium	-	-
DKMPPD2	[c]dkmpp + (3) h2o --> 2kmb + for + (6) h + pi	-	Glucose	Ammonium	-	-
DNMPPA	[c]dhpmp + h2o --> dhnpt + pi	-	Glucose	Ammonium	Oxygen	-
DPCOAK	[c]atp + dpcoa --> adp + coa + h	-	Glucose	Ammonium	-	-
FFSD	[c]h2o + suc6p --> fru + g6p	-	-	Ammonium	-	Sucrose
G5SADs	[c]glu5sa --> 1pyr5c + h + h2o	-	Glucose	Ammonium	Oxygen	-
KDOPP	[c]h2o + kdo8p --> kdo + pi	-	Glucose	Ammonium	Oxygen	-
MDRPD	[c]5mdru1p --> dkmpp + h2o	-	Glucose	Ammonium	Oxygen	-
MME	[c]mmcoa-R <==> mmcoa-S	b0004 b1479 b2463 b3403 b4383	Deoxyuridine	Nitrate	Fumarate	Glycine
MTRI	[c]5mdr1p <==> 5mdru1p	-	Glucose	Ammonium	Oxygen	-
MTRK	[c]5mtr + atp --> 5mdr1p + adp + h	-	Glucose	Ammonium	Oxygen	-
NADDPe	[e]h2o + nad --> amp + (2) h + nmn	b0750	Glucose	Ammonium	Oxygen	-
PEPT_EC	[c]cmp + h + (0.02) pe_EC <==> (0.02) 12dgr_EC + cdpea	-	Glucose	Ammonium	Oxygen	-
PGL	[c]6pgl + h2o --> 6pgc + h	b4025	Glucose	Ammonium	-	-
PMDPHT	[c]5aprbu + h2o --> 4r5au + pi	-	Glucose	Ammonium	-	-
PPCDC	[c]4ppcys + h --> co2 + pan4p	-	Glucose	Ammonium	-	-
PPNCL2	[c]4ppan + ctp + cys-L --> 4ppcys + cmp + h + ppi	-	Glucose	Ammonium	-	-
PPTGS	[c]uaagmda --> h + peptido_EC + udcpdp	-	Glucose	Ammonium	Oxygen	-
PTRCTA	[c]akg + ptrc --> 4abutn + glu-L	-	Acetaldehyde	Putrescine	Oxygen	-
SDPTA	[c]akg + sl26da <==> glu-L + sl2a6o	-	Glucose	Ammonium	Oxygen	-
SSALx	[c]h2o + nad + sucsal --> (2) h + nadh + succ	b2661	-	Ammonium	Oxygen	Putrescine
UDCPDP	[c]h2o + udcpdp --> h + pi + udcpp	-	Glucose	Ammonium	Oxygen	-
UNK3	[c]2kmb + glu-L --> akg + met-L	-	Glucose	Ammonium	Oxygen	-

Table S3. Biomass Components Produced by Glucose or Xylose. Under the proposed genetic conditions, the co-utilizing strain is incapable of growth when the media is composed entirely of glucose or xylose due to an inability to produce metabolites that make up the biomass. A list of what can and can not be produced by the sugars individually is provided in tabular form below. Metabolites highlighted in red indicate biomass components that cannot be produced from minimal media with either carbon source separately. Abbreviations match those found in iJO1366.

	Carbon Source	
	**Glucose**	**Xylose**
**Created Biomass Components**	UDCPDP, 10fthf, 2fe2s, 2ohph, 4fe4s, ala-L, amet, arg-L, asn-L, asp-L, atp, bmocogdp, btn, ca2, cl, coa, cobalt2, ctp, cu2, cys-L, datp, dctp, dgtp, dttp, fad, fe2, fe3, gln-L, glu-L, gly, gtp, h2o, his-L, ile-L, k, leu-L, lys-L, met-L, mg2, mlthf, mn2, mobd, nh4, ni2, phe-L, pheme, pro-L, pydx5p, ribflv, ser-L, sheme, so4, thf, thmpp,thr-L, trp-L, tyr-L, utp, val-L, zn2	ca2, cl, cobalt2, cu2, fe2, fe3, h2o, k, mg2, mn2, mobd, nh4, ni2, so4, zn2
**Unmet Biomass Components**	kdo2lipid4_e, murein5px4p_p, nad, nadp, pe160, pe160_p, pe161, pe161_p	UDCPDP, 10fthf, 2fe2s, 2ohph, 4fe4s, ala-L, amet, arg-L, asn-L, asp-L, atp, bmocogdp, btn, coa, ctp, cys-L, datp, dctp, dgtp, dttp, fad, gln-L, glu-L, gly, gtp, his-L, ile-L, kdo2lipid4_e, leu-L, lys-L, met-L, mlthf, murein5px4p_p, nad, nadp, pe160, pe160_p, pe161, pe161_p, phe-L, pheme, pro-L, pydx5p, ribflv, ser-L, sheme, thf, thmpp, thr-L, trp-L, tyr-L, utp, val-L

Table S4. Comparison of GPR Complexity between iJR904 and iJO1366. The difficulty in obtaining coupling in the larger iJO1366 *E. coli* model is in part due to the increase in gene promiscuity in the network.

Genome Network:	iJR904	iJO1366
Blocked Reactions:	178/ 931 (19%)	259/2255 (11%)
Reactions with Isozymes:	149/ 931 (16%)	652/2255 (29%)
One-to-One Relations:	342/ 904 (38%)	430/1366 (31%)
Subunit/Protein Complex Relations:	288/ 904 (32%)	475/1366 (35%)
Average # of Genes per Reaction:	1.57	1.85
Average # of Reactions per Gene:	1.61	3.06
Average # of Enzymes per Reaction:	1.14	1.52
Average # of Isozymes per Reaction with Multiple Enzymes:	2.26	3.02

Table S5. Average Time Needed to Find A FOCAL Solution Across the Various Networks. The time reported represents the average time needed to find a solution or, in the case where no solution is found, the time required to prove there is no solution or to time out searching. Media Coupled solutions are those solutions that do not require a gene to be deleted, but can be coupled solely by modifying the media composition. The Media+Deletion Coupled solutions are those that involve a gene being deleted while the Uncoupled solutions are those for which FOCAL could not find a solution. The All Solutions category represents the average time used when all reactions are considered. For comparisons, it took ~30 minutes for another bilevel optimization approach (OptORF) to find a five deletion strategy for ethanol production with iJR904. This time was reduced to ~12 seconds when bounds on the dual variables were also imposed [2].

**Time Statistics (min)**	Organism:			
Type of FOCAL Solution::	*B. subtillis*	*P. putida*	*E. coli* (iJR904)	*E. coli* (iJO1366)
Media Coupled	0.76	1.24	0.72	5.67
Media+Deletion Coupled	8.98	12.23	19.26	18.48
Uncoupled	172.64	149.00	167.02	170.81
All Solutions	23.25	24.58	31.79	75.64

3. References

1. Burgard AP, Nikolaev EV, Schilling CH, Maranas CD: **Flux Coupling Analysis of Genome-Scale Metabolic Network Reconstructions.** *Genome Research* 2004, **14:**301-312.
2. Kim J, Reed JL, Maravelias CT: **Large-Scale Bi-Level Strain Design Approaches and Mixed-Integer Programming Solution Techniques.** *PLoS ONE* 2011, **6:**e24162.
